# Effect of physical activity and exercise on endometriosis-associated symptoms: a systematic review

**DOI:** 10.1186/s12905-021-01500-4

**Published:** 2021-10-09

**Authors:** Merete Kolberg Tennfjord, Rakel Gabrielsen, Tina Tellum

**Affiliations:** 1 School of Health Sciences, Kristiania University College, Prinsensgate 7-9, 0152 Oslo, Norway; 2 Department of Obstetrics and Gynecology, Akershus University Hospital, Sykehusveien 25, 1478 Nordbyhagen, Norway; 3 Tollbugata Fysioterapi, Tollbugata 13, 3044 Drammen, Norway; 4 Department of Gynecology, Oslo University Hospital, PB 0424, 0459 Nydalen, Oslo, Norway

**Keywords:** Physiotherapy, Pelvic pain, Endometriosis, Physical activity, Exercise, Quality of life

## Abstract

**Background:**

Endometriosis is a common benign gynecological disease that has the potential to debilitate due to pain and reduced quality of life. Treatment modalities such as hormones and surgery have limitations and do not treat all dimensions of the problems caused by endometriosis, and physical activity (PA) and exercise have been suggested as alternative treatments. Aim of this study was to perform a systematic review and meta-analysis to assess the effect of PA and exercise on endometriosis-associated symptoms.

**Methods:**

Eleven databases were searched systematically. Study selection, quality assessment, and data extraction were carried out by two independent researchers in accordance with PRISMA guidelines. Eligibility criteria were women with diagnosed endometriosis receiving an intervention (PA and/or exercise). The primary outcome was pain intensity, but all outcomes were accepted.

**Results:**

This study screened 1045 citations for eligibility. Four interventional studies were identified, of which one showed fatal design flaws and so was excluded. Three studies, two randomized controlled trials (RCT) and one pre-post study with no control group, involving 109 patients were included in a descriptive synthesis. The interventions included flexibility and strength training, cardiovascular fitness, and yoga, and were performed from one to four times per week for a total duration of 8–24 weeks, with or without supervision. Only one study found improvements in pain intensity. One study showed decreases in stress levels. Due to the heterogeneity of the study outcomes and measures, as well as confounding factors, a quantitative meta-analysis could not be performed.

**Conclusion:**

The effect of PA and exercise as treatments for endometrioses-associated symptoms could not be determined due to significant limitations of the included studies. Future research should be based on RCTs of high methodological quality, measuring and reporting relevant core outcomes such as pain, improvements in symptoms and quality of life, and acceptability and satisfaction from the perspectives of patients. Furthermore, these outcomes need to be measured using reliable and validated tools.

***Trial registration number*:**

CRD42021233138.

**Supplementary Information:**

The online version contains supplementary material available at 10.1186/s12905-021-01500-4.

## Introduction

Endometriosis is a benign gynecological condition in which ectopic, endometrium-like cells are located outside of the uterine cavity [1]. The condition affects up to 10% of women of fertile age, with up to 70% being symptomatic [1, 2]. The main clinical symptom of endometriosis is severe pain during menstruation (dysmenorrhea) [1]. Pain during intercourse (dyspareunia) is also common, as well as the development of chronic pelvic pain (CPP) [1, 2]. Other conditions associated with endometriosis include irritable bowel syndrome, painful bladder syndrome, abdominal pain, migraine, loss of quality of life and fatigue [2–4]. It is hypothesized that a specific immunological and inflammatory pathway is common to all of these conditions and endometriosis [3, 5]. It takes a mean of 8 years to diagnose the endometriosis, during which musculoskeletal disorders secondary to endometriosis as well as psychological disorders may develop [6, 7].

There is no definite cure for endometriosis, and so the main focus of management is to control the associated pain, which is achieved by hormonal suppression of the disease or surgical excision [8]. Unfortunately, hormonal treatment can have intolerable side effects or become ineffective over time, while the effect of surgery is often short-lived [8]. Advances in the understanding of endometriosis have expanded the focus on less invasive and nonpharmacological treatments [8, 9]. International clinical guidelines have suggested focusing on the role of physical activity (PA) and exercise as part of the therapeutic approach for women suffering from endometriosis-associated symptoms [10]. The inflammation that defines endometriosis causes sensitization of pelvic organs and, ultimately leading to CPP [11]. This mechanism makes it plausible for the anti-inflammatory effect of PA and exercise to impede the development of the disease and ameliorate the associated pain [12].

PA and exercise were introduced for treating endometriosis-associated symptoms more than 3 decades ago [13]. However, these interventions have been studied mostly in terms of their ability to reduce the risk of developing endometriosis [12, 14, 15], and so little is known about the effect of PA and exercise on symptom improvement in women with endometriosis [12]. Some effect of PA and exercise has been found in women with CPP without endometriosis [16], but it is unclear whether this effect is transferable to women with endometriosis-associated pain [10].

Two previous systematic reviews have addressed the effect of PA and exercise on endometriosis-associated symptoms [17, 18]. However, these studies mainly focused on other complementary and alternative treatment options for endometriosis, such as mind–body interventions and acupuncture. The effect of PA and exercise specifically remained unclear since their searches were limited to a few databases, the search terms were not specified [18], or “PA” and “exercise” were not included as search terms [19]. This raises the possibility that relevant studies on this subject were overlooked.

The present systematic review attempted to identify interventional studies of high quality to assess the effect of PA and exercise specifically in treating women with endometriosis-associated symptoms.

## Review question

What is the effect of PA and exercise on endometriosis-associated symptoms?

## Methods

This systematic review was registered in the International Prospective Register of Systematic Reviews (CRD42021233138), and was performed in accordance with the PRISMA (Preferred Reporting Items for Systematic Reviews and Meta-Analyses) guidelines [20] (Additional file 1).

### Eligibility criteria and search strategy

Studies of interventions involving any type of PA and exercise were eligible for inclusion. PA was defined as “any bodily movement produced by skeletal muscles that requires energy expenditure” [21] and exercise was defined as “PA that is planned, structured, and repetitive for the purpose of conditioning the body” [21], consisting of cardiovascular conditioning, strength and resistance training, and flexibility.

The study population consisted of women with any degree of endometriosis as diagnosed with an imaging or surgical modality, who presented with pain in the pelvic region (including dysmenorrhea, dyspareunia, or CPP). The primary outcome measure was the pain intensity, but all outcomes were accepted.

Exclusion criteria were data presented in short communications, reviews, letters to the editor, and congress abstracts, and the application of passive interventions such as manual therapy to patients. The literature search was completed with support from a trained medical librarian. The search included the Cochrane Central Register of Controlled Trials, Embase, PubMed, MEDLINE, PsycInfo, CINAHL, AMED, Scopus, Web of Science, PEDro, and SveMed + , without time limitation up to December 2020. Publications could be in English, Swedish, Norwegian, Danish, or German. Search terms were identified through a pilot search for relevant literature. The electronic search strategy for this systematic review is presented in Additional file 2. In addition, the reference lists of included articles and identified reviews on the topic were scanned, and manually searched for further studies.

### Study selection and quality assessment

In the first step, all obtained references were independently screened on the basis of the title and Abstract by M.K.T. and T.T. using the Rayyan web application [22] that allows blinded assessments. In the second step, all Abstracts with conflicting decisions were reviewed by both authors until consensus was reached. In the third step, the same authors independently assessed the methodological quality of the manuscripts that met the inclusion criteria, using quality assessment questionnaires appropriate for the design of each study as provided by the National Heart Lung and Blood Institute [23]. We applied the method of “quality assessment of controlled intervention studies” for randomized controlled trials (RCT), and the “quality assessment of before-after (pre-post) studies with no control group” for intervention studies with no control group while adding relevant questions to determine exposure, risk, and confounding variables. The assessment tools include several criteria rated “yes,” “no,” and “other: CD, cannot determine; NA, not applicable; NR, not reported.” The quality of the included studies was rated as good, fair, or poor. We also used the Consensus on Exercise Reporting Template (CERT) [24], which is a 19-item checklist that yields a detailed description of the minimum criteria that should be reported in an exercise intervention. The template provides individual scores for each included article (ranging from 0 to 19), in addition to a summary score for each item.

### Data extraction

The full text of eligible articles was read by two reviewers (M.K.T. and T.T.), who independently extracted the following data: author(s), year of publication, study period, country of origin, study design, sample size, inclusion and exclusion criteria, intervention type, description of intervention, follow-up period, primary and secondary outcomes, and dropout rate.

### Data reporting and summary measures

A meta-analysis was planned, but it could not be performed due to the substantial heterogeneity found in study designs and outcomes. Results from the studies are reported as between- and within-group differences using mean ± standard-deviation values or numbers with percentages, according to availability. Probability values were rounded to two decimal places, with the exception of *p* < 0.001. Confidence intervals were provided if available.

## Results

### Study selection

**Fig. 1:**
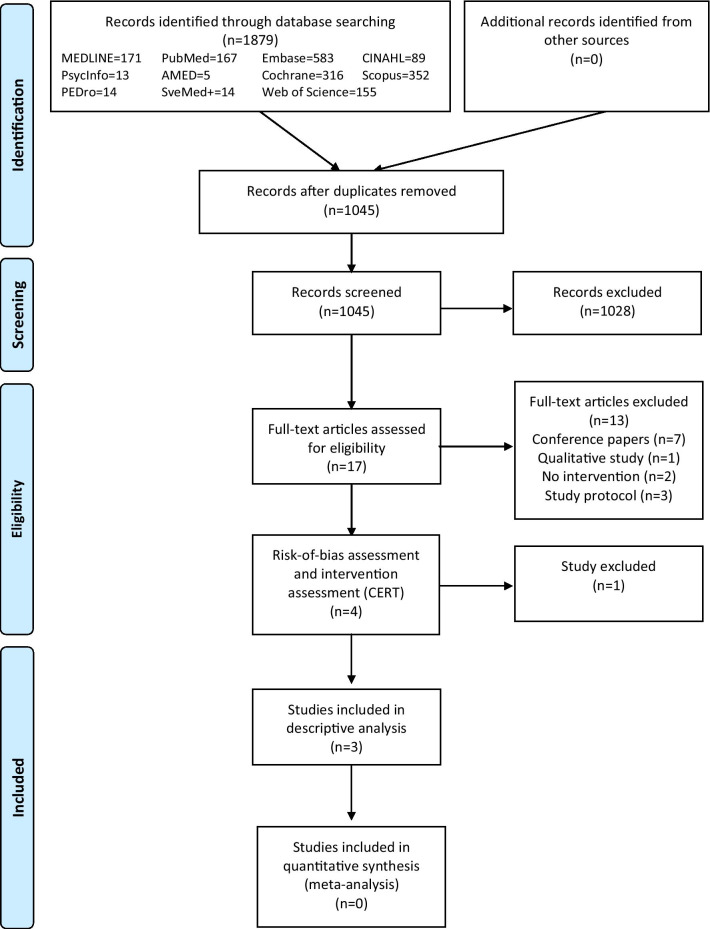
PRISMA (Preferred Reporting Items for Systematic Reviews and Meta-Analyses) flow diagram for the identification, screening, eligibility, and inclusion of relevant articles

This study identified 1879 citations (Fig. 1). After removing duplicates, the remaining 1045 citations were screened for eligibility based on the title and Abstract. Seventeen publications were assessed for further inclusion reading the full-text versions of the articles, and four publications were included for quality assessment [25–28]. We identified four studies that described an intervention incorporating PA and/or exercise: two were RCTs [27, 28] and two were pre-post studies with no control group [25, 26] (Tables 1, 2).

**Table 1:** Quality assessment of controlled intervention studies

References	Clearly stated study design	Randomization adequate	Treatment allocation concealed	Participant and providers blinded	Assessor blinded	Baseline characteristics similar	Dropout rate ≤ 20%	Differential dropout rate ≤ 15%
Goncalves et al. [28]	Yes	Yes	Yes	NA	NA	No	No	No
Carpenter et al. [27]	Yes	Yes	NR	NA	NR	Yes	Yes	Yes

References	High adherence to treatment	Other interventions	Outcomes valid and reliable	Sample size provided 80% power	Predefined outcome measures	Intention to treat	Confounding variables measured and adjusted	Quality rating (poor/fair/good)
Goncalves et al. [28]	No	No	Yes	Yes	Yes	Yes	No	Poor
Carpenter et al. [27]	Yes	Yes	Yes	No	Yes	Yes	No	Fair

*NA* not applicable, *NR* not reported. The item “confounding variables measured and adjusted” was added to the original assessment form

**Table 2:** Quality assessment for before-after (pre-post) studies with no control group

References	Question or objective stated	Eligible criteria pre-specified and described	Participants representative of clinical population	All eligible participants enrolled	Sample size sufficient	Intervention clearly described/delivered consistently	Outcomes prespecified /valid and reliable, and consistently implemented	Blinded assessors
Friggi Sebe Petrelluzzi et al. [25]	Yes	Yes	Yes	Yes	CD	Yes	Yes	NA
Awad et al. [26]	Yes	Yes	No	No	CD	Yes	Yes	NA

References	Dropout rate ≤ 20% or accounted for in the analysis	Statistics well described	Outcomes tested multiple times	Analysis of individual-level data	Confounding variables measured and adjusted	Exposure/risk defined/valid and reliable, and implemented consistently	Quality rating (poor/fair/good)
Friggi Sebe Petrelluzzi et al. [25]	Yes	Yes	no	NA	No	Yes	Poor
Awad et al. [26]	Yes	Yes	No	NA	No	No	Poor*

*CD* could not determine. Items “confounding variables measured and adjusted” and “exposure/risk defined/valid and reliable, and implemented consistently” was added to the original assessment form. *This study was excluded from the qualitative synthesis due to fatal flaws in its design

### Quality assessment, risk of bias, and exercise intervention assessment

One study was rated as being of fair quality [27], while three were rated as poor quality [25, 26, 28]. The detailed assessment including signaling questions are presented in Tables 1 and 2. The RCT of Carpenter et al. [27] was judged as being of fair quality (Table 1). The main limitation of that RCT for the purpose of this review was that the participants were treated with danazol, which is a potent drug for treating endometriosis. Though having a control group, the study was underpowered for determining whether exercise had an additional effect to danazol. However, since the study was designed to investigate if exercise could alleviate the side effects of danazol, it was not flawed per se. Moreover, the sample was too small to allow comparisons of individual side effects, important secondary outcomes (pelvic pain, dysmenorrhea, and dyspareunia) were not reported, and the methods of randomization and outcome assessment were not reported.

The RCT of Goncalves et al. [28] was judged as being of poor quality due to significant differences in the baseline characteristics between the intervention and control groups (Table 1). The intervention group had a higher level of education, a high percentage of homemakers, and a lower rate of employment, which is a confounder for quality-of-life assessments. Also, one of the inclusion criteria was the presence of therapy-resistant CPP, which is a possible confounder for endometriosis-associated symptoms. Furthermore, the control group also received physiotherapy. Finally, the dropout rate in the intervention group was very high, at 30% (vs 0% in the control group).

The study of Friggi Sebe Petrelluzzi et al. [25] was judged as being of fair quality (Table 2). As in Goncalves et al. [28] only women with endometriosis and therapy-resistant CPP were included, representing a confounder. Furthermore, a sample-size calculation was not reported, and there was no control group. The intervention consisted not only of PA and exercise, but also a range of modalities including behavioral cognitive therapy, which confounds the contribution of PA and exercise to symptom improvement.

The study of Awad et al. [26] was judged as being of poor quality (Table 2). It was ultimately excluded from the synthesis since its design was fatally flawed by initiating medroxyprogesterone acetate, an effective hormonal treatment for endometriosis, at the same time as the intervention but without including a control group. Furthermore, no sample-size calculation was provided, and the inclusion and exclusion criteria appeared to be random from a clinical perspective.

The individual scores for the articles based on the CERT checklist (Additional file 3) ranged from 7 to 14. None of the articles provided a description of exercise progression [25–28], and only one included a description of individually tailored exercises [27]. Exercise adherence was measured adequately in one study [27], as were motivational strategies [25]. The level of exercise was only described for two studies [27, 28].

### Study populations

The total study sample consisted of 109 participants [25, 27, 28] (Table 3). Two studies included women with surgically confirmed endometriosis [25, 27], while it was not specified how endometriosis was diagnosed by Goncalves et al. [28]. The stage of endometriosis was not reported for any of the studies. The age of the included women was provided for two studies [25, 28]. All of the women in two studies [25, 28] also had CPP. Details of prior hormonal or surgical treatments were not provided for any of the studies.

**Table 3:** Characteristics of the included studies

References	Country	Study period	Study design	Number	Study population	Intervention description	Control group	Duration	Primary outcome (measure)	Secondary outcomes (measure)	Dropouts, n (%)
Carpenter et al. [27]	USA	NR	RCT	39 (18 intervention vs 18 controls)	Endometriosis^1^ with no other hormonal treatment during previous 12 months, no regular exercise	Unsupervised; 40 min of individualized cardio fitness at 50–70% of max heart rate + flexibility exercises + danazol	Danazol treatment only	Four times weekly for 24 weeks	Number of side effects of danazol (direct inquiry)	Fitness (VO2max), general muscle strength (KINCOM), sex hormone levels, pelvic symptoms	3 (7.69%), only in control group
Friggi Sebe Petrelluzzi et al. [25]	Brazil	NR	Pre-post, no control group	30	Women with endometriosis^1^ and ≥ 7 years of CPP, with no effect of medical therapy or surgery, age ^2^32.0 ± 1.30 years	Supervised; 1 h of body awareness, breathing exercise, stretching, general movement, PFM strength + 1.5 h behavioral cognitive therapy	No control group	1.0 to 1.5 h for 10 weeks	Pain (VAS, 0–10)	Stress (PSQ), QOL (SF-36), salivary cortisol levels	4 (13.33%)
Goncalves et al. [28]	Brazil	08/2013 to 12/2014	RCT	40 (28 intervention vs 12 controls)	Endometriosis^3^ and CPP, prior hormonal and surgical therapy, age ^2^34.88 ± 6.70 years, no regular exercise	Supervised; 120 min of Hatha yoga, including posture (60 min) + conversation (30 min) + relaxation, breathing exercises, meditation (30 min) Medical therapy was continued	Continuing medical therapy or physiotherapy once per week	Twice weekly for 8 weeks	QOL (EHP-30)	Pain (VAS, 0–10), menstrual pattern measured daily (amount of bleeding scored from 0 to 5)	12 (30%), only in intervention group

^1^Confirmed by laparoscopy; ^2^mean ± standard deviation; ^3^not specified how diagnosed; *NR* not reported, *QOL* quality of life, *RCT* randomized controlled trial, *PFM* pelvic floor muscles, *CPP* chronic pelvic pain, *VAS* visual analogue scale, *KINCOM* Kinetic Communicator Exercise System, *PSQ* Perceived Stress Questionnaire, *SF-36* 36-item Short-Form Health Survey, *EHP-30* Endometriosis Health Profile-30

### Interventions

The performed interventions are listed in Table 1. No study performed a follow-up after the intervention had finished. Confounding interventions to PA and exercise were identified in all studies, as explained above. Limitations in the reporting of exercise interventions (according to the CERT) are also explained above.

### Primary and secondary outcome measures

The primary and secondary outcomes for all studies are reported in Table 3. Only one study had “pain” as the primary outcome [25]. The outcome reports were incomplete for all studies.

### Effect of intervention on pain

Goncalves et al. [28] reported that the degree of daily pain was significantly lower in the intervention group than the control group, although the difference in the mean scores on a visual analogue scale (VAS) was not provided (*p* < 0.001). Furthermore, the scores in pain-related domains on Endometriosis Health Profile-30 (EHP-30) were significantly lower in the yoga group than the control group at postintervention (32.39 ± 21.95 versus 55.05 ± 21.49, *p* < 0.001). Notably, the control group also received physiotherapy following the intervention at their institution.

Friggi Sebe Petrelluzzi et al. [25] did not find a significant improvement in pain intensity (change in VAS score from pre- to posttreatment: 4.00 ± 0.56 to 3.30 ± 0.65, *p* > 0.05). Carpenter et al. [27] found that the pelvic pain decreased in both the intervention and control groups, with medical treatment using danazol providing no additional effect relative to that obtained by PA and exercise. However, the exact results and significance level were not reported, leaving it uncertain about whether a type II error was present due to the sample being too small.

### Effects of intervention on mental health aspects and well-being

The study of Friggi Sebe Petrelluzzi et al. [25] measured stress levels using the Perceived Stress Questionnaire (PSQ), salivary cortisol levels, and the 36-item Short-Form Health Survey (SF-36). The PSQ was developed as an outcome measure in psychosomatic research and was validated for use in Brazil [29]. Perceived stress was significantly lower at pretreatment (0.62 ± 0.02) than posttreatment (0.56 ± 0.02, *p* < 0.05). Significant improvements in the vitality and physical functioning domains of the SF-36 were also found (*p* < 0.05), but these effects were no longer significant after performing a multivariate analysis that included each variable in the SF-36. There was an overall decrease in salivary cortisol levels from pretreatment to posttreatment (*p* = 0.04), but this was not correlated with perceived stress as measured with PSQ. Goncalves et al. [28] found significant improvements in certain EHP-30 items (control and powerlessness, emotional well-being, and self-image) in the intervention group compared with the control group (*p* < 0.001).

### Effect of intervention on pelvic floor dysfunction

The study of Carpenter et al. [27] assessed how exercise during danazol treatment could improve pelvic floor symptoms such as dyspareunia and dysmenorrhea. Those authors reported that the symptoms improved in both groups, but the values and significance levels were not provided. Goncalves et al. [28] found that the sexual-intercourse domain of EHP-30 was lower after 8 weeks of Hatha yoga in both the intervention and control groups, but the result did not reach between- or within-groups significance.

## Discussion

This systematic review has summarized the available evidence for the effect of PA and exercise on endometriosis-associated symptoms. We identified 4 interventional studies involving 129 women. However, 1 of these studies was excluded after the quality assessment revealed fatal flaws in its design, leaving 109 women being finally included. Each included study found some improvement in pain intensity, stress levels, well-being, or self-image. However, due to confounding factors, the effect of PA and exercise alone could not be determined. Furthermore, the heterogeneity of the outcome measures and incomplete outcome reporting made it impossible to conduct a quantitative meta-analysis.

The relationship of PA and exercise with endometriosis has been widely studied in the past, and several reviews have been published on this topic [12, 14, 15, 18, 19]. However, their results have been inconclusive, mainly due to inclusion of observational studies of how PA and exercise may lower the risk of developing endometriosis [12, 14, 15]. Another possible reason for the inconclusive findings is the diversity in the type of interventions included in other systematic reviews [18, 19], where the focus has spanned from acupuncture and yoga to electrotherapy and exercise.

A multimodal approach that includes physiotherapy has been suggested to alleviate endometriosis symptoms [10, 30, 31]. Physiotherapy contains both active and passive modalities, but the optimal physiotherapy approach for endometriosis-associated symptoms is not clear [16]. The theory supporting PA and exercise as a beneficial approach involves viewing the skeletal muscles as an endocrine organ, with contraction of these muscles releasing myokines [32]. These myokines may exert direct effects on the muscle itself or distal organs such as the liver, pancreas, or adipose tissue [32]. Furthermore, exercise increases the production of leucocytes, cortisol, and adrenaline, all of which have potent acute anti‐inflammatory effects [33].

The present review specifically focused on PA and exercise, but it was not possible to summarize the effect due to significant limitations of the included studies. However, some trends could be identified. One study showed improvements in daily pain scores [28], but no effect-size measures were provided, and so the strength of this association was uncertain. Furthermore, the effect of Hatha yoga was questionable due to the additional time spent on relaxation and meditation [28]. A recent systematic review and meta-analysis produced evidence that meditation itself is effective in improving the quality of life and pain in women with CPP [34], which was an inclusion criterion in the present study. However, the two other studies in our review did not find an effect from PA and exercise on pain [25, 27]. No sample-size calculations were performed for those two studies, and so type II errors might have been present.

There seems to be a dose–response relationship between regular, high-intensity exercise and the effect on the inflammatory profile in general [33]. Since none of the studies in this review included descriptions of exercise progression [25, 27, 28] (Additional file 3), we can only speculate if the effect of PA and exercise would have been stronger if progressive overload had been achieved [24]. Other reported effects were reduced stress levels by Friggi Sebe Petrelluzzi et al. [25], and improvements in well-being and body image by Goncalves et al. [28]. Both of these studies included women with CPP and applied a cognitive approach in addition to PA and exercise, which are both possible confounders for the effect of PA and exercise on endometriosis-associated symptoms [34].

Previous research has found that the pelvic floor muscle tension in higher in women suffering from endometriosis pain [35] than in controls without endometriosis. Since a large proportion of women with endometriosis suffer from dyspareunia and CPP [1, 2], it is surprising that only one of the present studies investigated the pelvic floor muscles [25]; however, pain scores specifically for the pelvic floor or measurements of the pelvic floor muscles were not reported. Lastly, none of the studies measured patient satisfaction. The high dropout rate found by Goncalves et al. [28] indicates that it is pertinent to design exercise interventions that meet the needs of patients and fit their lifestyle.

In a recent initiative, healthcare professionals and women suffering from endometriosis were able to recommend a minimum set of outcomes to be measured and reported in all interventional clinical trials of endometriosis [36]. Those so-called core outcomes aim to focus research on meaningful endpoints for the users of health services [37]. Patient satisfaction with the treatment was one of those outcomes. There are several ongoing RCTs related to PA and exercise [38–40] that are measuring the following core items: pain, improvement in symptoms and quality of life, patient acceptability, and patient satisfaction with the treatment. These trials might yield evidence-based advice on PA and exercise for women with endometriosis-associated symptoms in the future.

### Strength and limitations

The strengths of this systematic review include its originality, rigorous search strategy, and methodological robustness. Its main limitation is the low grade of evidence that could be obtained from the previous studies. The small samples, confounding factors, heterogeneity of interventions, and poor reporting of details about the exercise intervention and outcome measures restricts our ability to draw overall conclusions about the effect of PA and exercise in treating endometriosis-associated symptoms.

## Conclusion

PA and exercise might exert a range of beneficial effects on endometriosis-associated symptoms, but unfortunately these effects cannot be robustly determined based on the existing literature. Nevertheless, the potentially beneficial role of PA and exercise should be communicated to women with endometriosis-associated symptoms. Future research should be based on RCTs of high methodological quality, measuring and reporting relevant core outcomes such as pain, improvements in symptoms and quality of life, and acceptability and satisfaction from the perspectives of patients. Furthermore, these outcomes need to be measured using reliable and validated tools. A focus on the type and dose of PA and exercise as well as patient selection is warranted, and using appropriate checklists such as the CERT is recommended. Since endometriosis patients can show complex symptomatology, the cooperation of multiple disciplines such as physiotherapists and gynecologists could improve the quality of clinical research in this field.

## Supplementary Information

**Additional file 1. **PRISMA (Preferred Reporting Items for Systematic Reviews and Meta-Analyses).

**Additional file 2**. Electronic search strategy with search terms.

**Additional file 3**. CERT (Consensus on Exercise Reporting Template).

## Data Availability

Not applicable. Relevant material is attached as Additional files.

## Abbreviations

PA: Physical activity
RCT: Randomized controlled trials
CPP: Chronic pelvic pain
PRISMA: Preferred Reporting Items for Systematic Reviews and Meta-Analyses
CERT: Consensus on Exercise Reporting Template
VAS: Visual analogue scale
EHP-30: Endometriosis Health Profile-30
*P*: *P*-value
PSQ: Perceived Stress Questionnaire
SF-36: 36-Item Short-Form Health Survey
