# Supplementary material for: Effect of physical activity and exercise on endometriosis-associated symptoms: a systematic review
Source: BMC Womens Health. 2021 Oct 9;21:355. doi: 10.1186/s12905-021-01500-4 (PMC8502311; doi:10.1186/s12905-021-01500-4)
Supplement: Supplementary file 2 — Additional file 2. Electronic search strategy with search terms. [file 12905_2021_1500_MOESM2_ESM.docx]

Supplementary Material 2. Electronic search strategy with search terms

Date for search: 15-16 December 2020
Information specialist: Åse Marit Hammersbøen
Total number of hits from bibliographic databases: **1879**
Total number of hits after removing duplicates: **1045**

Database: **Ovid MEDLINE(R) ALL**1946 to December 15, 2020

| **#** | **Searches** | **Results** |
| --- | --- | --- |
| 1 | Endometriosis/ | 22138 |
| 2 | endometrio*.ti,ab,kf. | 31775 |
| 3 | 1 or 2 | 35160 |
| 4 | exp Pain/ | 402146 |
| 5 | pain*.ti,ab,kf. | 719383 |
| 6 | 4 or 5 | 867976 |
| 7 | exp Exercise/ or exp Physical Therapy Modalities/ or exp Exercise Movement Techniques/ or exp Exercise Therapy/ or exp Sports/ | 422305 |
| 8 | (exercis* or physical-activ* or sport* or physical-therap* or physiotherap* or physio-therap* or movement-therap* or motion-therap* or kinesiotherap* or kinesitherap* or strength* or jog* or walk* or breathing-exercis* or yoga* or yogi* or pilates* or aerobic* or qigong* or qi-gong* or tai-ji* or taji* or tai-chi* or danc* or gymnast* or bicycl* or plyometric* or stretch* or swim* or workout* or work-out* or ((aerob* or anaerob* or circuit* or endurance* or resistance* or interval* or high-intens* or moderat* or low-intens*) adj3 (exercise* or train*)) or (non-surgical* adj (management* or intervention*))).ti,ab,kf,hw. | 1316204 |
| 9 | 7 or 8 | 1408605 |
| 10 | 3 and 6 and 9 | 171 |

Database: **Embase**1974 to 2020 December 15

| **#** | **Searches** | **Results** |
| --- | --- | --- |
| 1 | Endometriosis/ | 37938 |
| 2 | endometrio*.ti,ab,kw. | 46781 |
| 3 | 1 or 2 | 53139 |
| 4 | exp Pain/ | 1354442 |
| 5 | pain*.ti,ab,kw. | 1056763 |
| 6 | 4 or 5 | 1707801 |
| 7 | exp Physical activity/ or exp Exercise/ or exp Kinesiotherapy/ or exp Physiotherapy/ or exp Sport/ | 881871 |
| 8 | (exercis* or physical-activ* or sport* or physical-therap* or physiotherap* or physio-therap* or movement-therap* or motion-therap* or kinesiotherap* or kinesitherap* or strength* or jog* or walk* or breathing-exercis* or yoga* or yogi* or pilates* or aerobic* or qigong* or qi-gong* or tai-ji* or taji* or tai-chi* or danc* or gymnast* or bicycl* or plyometric* or stretch* or swim* or workout* or work-out* or ((aerob* or anaerob* or circuit* or endurance* or resistance* or interval* or high-intens* or moderat* or low-intens*) adj3 (exercise* or train*)) or (non-surgical* adj (management* or intervention*))).ti,ab,kw,hw. | 1773064 |
| 9 | 7 or 8 | 1907888 |
| 10 | 3 and 6 and 9 | 583 |

Database: **APA PsycInfo**1806 to December Week 1 2020

| **#** | **Searches** | **Results** |
| --- | --- | --- |
| 1 | endometrio*.ti,ab,id. | 304 |
| 2 | exp Pain/ | 58774 |
| 3 | pain*.ti,ab,id. | 113007 |
| 4 | 2 or 3 | 124622 |
| 5 | exp Exercise/ or Movement Therapy/ or Physical Therapy/ or exp Sports/ | 63628 |
| 6 | (exercis* or physical-activ* or sport* or physical-therap* or physiotherap* or physio-therap* or movement-therap* or motion-therap* or kinesiotherap* or kinesitherap* or strength* or jog* or walk* or breathing-exercis* or yoga* or yogi* or pilates* or aerobic* or qigong* or qi-gong* or tai-ji* or taji* or tai-chi* or danc* or gymnast* or bicycl* or plyometric* or stretch* or swim* or workout* or work-out* or ((aerob* or anaerob* or circuit* or endurance* or resistance* or interval* or high-intens* or moderat* or low-intens*) adj3 (exercise* or train*)) or (non-surgical* adj (management* or intervention*))).ti,ab,id,hw. | 312485 |
| 7 | 5 or 6 | 318300 |
| 8 | 1 and 4 and 7 | 13 |

Database: **Cochrane Central Register of Controlled Trials (CENTRAL)**

| **ID** | **Search** | **Hits** |
| --- | --- | --- |
| #1 | MeSH descriptor: [Endometriosis] this term only | 856 |
| #2 | (endometrio*):ti,ab,kw | 2890 |
| #3 | #1 or #2 | 2890 |
| #4 | MeSH descriptor: [Pain] explode all trees | 49481 |
| #5 | (pain*):ti,ab,kw | 185873 |
| #6 | #4 or #5 | 192016 |
| #7 | MeSH descriptor: [Exercise] explode all trees | 24606 |
| #8 | MeSH descriptor: [Physical Therapy Modalities] explode all trees | 25626 |
| #9 | MeSH descriptor: [Exercise Movement Techniques] explode all trees | 2124 |
| #10 | MeSH descriptor: [Exercise Therapy] explode all trees | 13799 |
| #11 | MeSH descriptor: [Sports] explode all trees | 15588 |
| #12 | (exercis* OR "physical activ*" OR sport* OR "physical therap*" OR physiotherap* OR "physio therap*" OR "movement therap*" OR "motion therap*" OR kinesiotherap* OR kinesitherap* OR strength* OR jog OR jogging* OR walk* OR "breathing exercis*" OR yoga* OR yogi* OR pilates* OR aerobic* OR qigong* OR "qi gong*" OR "tai ji*" OR taji* OR "tai chi*" OR danc* OR gymnast* OR bicycl* OR plyometric* OR stretch* OR swim* OR workout* OR (work NEXT out*)):ti,ab,kw | 163693 |
| #13 | (((aerob* OR anaerob* OR circuit* OR endurance* OR resistance* OR interval* OR (high NEXT intens*) OR moderat* OR (low NEXT intens*)) NEAR/3 (exercise* OR train*))):ti,ab,kw | 29491 |
| #14 | ("non-surgical" NEAR/1 (management* OR intervention)):ti,ab,kw | 365074 |
| #15 | {OR #7-#14} | 473855 |
| #16 | #3 AND #6 AND #15 | 335 |
|  | **Referanser i CENTRAL** | **316** |

Database: **PubMed**

(Endometriosis[mesh] OR endometrio*[tiab]) AND (Pain[mesh] OR pain*[tiab]) AND (Exercise[Mesh] OR "Physical Therapy Modalities"[mesh] OR "Exercise Movement Techniques"[Mesh] OR exp "Exercise Therapy"[mesh] OR exp Sports[mesh] OR exercis*[tiab] OR physical-activ*[tiab] OR sport*[tiab] OR physical-therap*[tiab] OR physiotherap*[tiab] OR physio-therap*[tiab] OR movement-therap*[tiab] OR motion-therap*[tiab] OR kinesiotherap*[tiab] OR kinesitherap*[tiab] OR strength*[tiab] OR jog[tiab] OR jogging*[tiab] OR walk*[tiab] OR breathing-exercis*[tiab] OR yoga*[tiab] OR yogi*[tiab] OR pilates*[tiab] OR aerobic*[tiab] OR qigong*[tiab] OR qi-gong*[tiab] OR tai-ji*[tiab] OR taji*[tiab] OR tai-chi*[tiab] OR danc*[tiab] OR gymnast*[tiab] OR bicycl*[tiab] OR plyometric*[tiab] OR stretch*[tiab] OR swim*[tiab] OR workout*[tiab] OR work-out*[tiab] OR ((aerob*[tiab] OR anaerob*[tiab] OR circuit*[tiab] OR endurance*[tiab] OR resistance*[tiab] OR interval*[tiab] OR high-intens*[tiab] OR moderat*[tiab] OR low-intens*[tiab]) AND (exercise*[tiab] OR train*[tiab])) OR (non-surgical*[tiab] AND (management*[tiab] OR intervention*[tiab])))

**Søkeresultat: 167 referanser**

Database: **Cinahl** (Cumulative Index to Nursing and Allied Health Literature)

| **#** | **Query** | **Results** |
| --- | --- | --- |
| S1 | (MH "Endometriosis") | 4,695 |
| S2 | TI endometrio* OR AB endometrio* | 6,990 |
| S3 | S1 OR S2 | 7,799 |
| S4 | (MH "Pain+") | 208,607 |
| S5 | TI pain* OR AB pain* | 268,842 |
| S6 | S4 OR S5 | 340,878 |
| S7 | MH ("physical activity" OR exercise+ OR "physical therapy+" OR "therapeutic exercise+" OR sports+) | 258,962 |
| S8 | TI ( exercis* OR “physical activ*” OR sport* OR “physical therap*” OR physiotherap* OR “physio therap*” OR “movement therap*” OR “motion therap*” OR kinesiotherap* OR kinesitherap* OR strength* OR jog* OR walk* OR “breathing exercis*” OR yoga* OR yogi* OR pilates* OR aerobic* OR qigong* OR “qi gong*” OR “tai ji*” OR taji* OR “tai chi*” OR danc* OR gymnast* OR bicycl* OR plyometric* OR stretch* OR swim* OR workout* OR work-out* OR ((aerob* OR anaerob* OR circuit* OR endurance* OR resistance* OR interval* OR “high-intens*” OR moderat* OR “low-intens*”) N3 (exercise* OR train*)) OR (“non-surgical*” N1 (management* OR intervention*)) ) OR AB (exercis* OR “physical activ*” OR sport* OR “physical therap*” OR physiotherap* OR “physio therap*” OR “movement therap*” OR “motion therap*” OR kinesiotherap* OR kinesitherap* OR strength* OR jog* OR walk* OR “breathing exercis*” OR yoga* OR yogi* OR pilates* OR aerobic* OR qigong* OR “qi gong*” OR “tai ji*” OR taji* OR “tai chi*” OR danc* OR gymnast* OR bicycl* OR plyometric* OR stretch* OR swim* OR workout* OR work-out* OR ((aerob* OR anaerob* OR circuit* OR endurance* OR resistance* OR interval* OR “high-intens*” OR moderat* OR “low-intens*”) N3 (exercise* OR train*)) OR (“non-surgical*” N1 (management* OR intervention*)) ) | 394,614 |
| S9 | S7 OR S8 | 545,357 |
| S10 | S3 AND S6 AND S9 | 89 |

Database: **AMED** (Allied and Complementary Medicine) 1985 to November 2020

| **#** | **Searches** | **Results** |
| --- | --- | --- |
| 1 | endometrio*.ti,ab,hw. | 130 |
| 2 | pain*.ti,ab,hw. | 34967 |
| 3 | (exercis* or physical-activ* or sport* or physical-therap* or physiotherap* or physio-therap* or movement-therap* or motion-therap* or kinesiotherap* or kinesitherap* or strength* or jog* or walk* or breathing-exercis* or yoga* or yogi* or pilates* or aerobic* or qigong* or qi-gong* or tai-ji* or taji* or tai-chi* or danc* or gymnast* or bicycl* or plyometric* or stretch* or swim* or workout* or work-out* or ((aerob* or anaerob* or circuit* or endurance* or resistance* or interval* or high-intens* or moderat* or low-intens*) adj3 (exercise* or train*)) or (non-surgical* adj (management* or intervention*))).ti,ab,hw. | 73176 |
| 4 | 1 and 2 and 3 | 5 |

Database: **Scopus**

TITLE-ABS-KEY ( endometrio* AND pain* AND ( exercis* OR "physical activ*" OR sport* OR "physical therap*" OR physiotherap* OR "physio therap*" OR "movement therap*" OR "motion therap*" OR kinesiotherap* OR kinesitherap* OR strength* OR jog OR jogging* OR walk* OR "breathing exercis*" OR yoga* OR yogi* OR pilates* OR aerobic* OR qigong* OR "qi gong*" OR "tai ji*" OR taji* OR "tai chi*" OR danc* OR gymnast* OR bicycl* OR plyometric* OR stretch* OR swim* OR workout* OR "work-out*" OR ( ( aerob* OR anaerob* OR circuit* OR endurance* OR resistance* OR interval* OR "high-intens*" OR moderat* OR "low-intens*" ) W/3 ( exercise* OR train* ) ) OR ( "non-surgical*" W/1 ( management* OR intervention ) ) ) )

**Søkeresultat: 352 treff**

Database: **Web of Science**

TS=(endometrio*) AND TS=(pain*) AND TS=(exercis* OR physical-activ* OR sport* OR physical-therap* OR physiotherap* OR physio-therap* OR movement-therap* OR motion-therap* OR kinesiotherap* OR kinesitherap* OR strength* OR jog OR jogging* OR walk* OR breathing-exercis* OR yoga* OR yogi* OR pilates* OR aerobic* OR qigong* OR qi-gong* OR tai-ji* OR taji* OR tai-chi* OR danc* OR gymnast* OR bicycl* OR plyometric* OR stretch* OR swim* OR workout* OR work-out* OR ((aerob* OR anaerob* OR circuit* OR endurance* OR resistance* OR interval* OR high-intens* OR moderat* OR low-intens*) NEAR/3 (exercise* OR train*) ) OR (non-surgical* NEAR/1 (management* OR intervention) ))

*Indexes=SCI-EXPANDED, SSCI, A&HCI, ESCI Timespan=1945-2020*

**Søkeresultat: 155 treff**

Database: **PEDro**

endometriosis AND pain

**Søkeresultat: 14 referanser**

Database: **SveMed+**

endometriosis AND pain

**Søkeresultat: 14 referanser**
