# Supplementary material for: Effect of physical activity and exercise on endometriosis-associated symptoms: a systematic review
Source: BMC Womens Health. 2021 Oct 9;21:355. doi: 10.1186/s12905-021-01500-4 (PMC8502311; doi:10.1186/s12905-021-01500-4)
Supplement: Supplementary file 3 — Additional file 3. CERT (Consensus on Exercise Reporting Template). [file 12905_2021_1500_MOESM3_ESM.docx]

Supplementary Material 3. CERT (Consensus on Exercise Reporting Template).

| Authors, year | 1. Equipment | 2. Instructor qualifications | 3. Individual or group | 4. Supervision | 5. Adherence | 6. Motivation strategies | 7a. Rules for progression | 7b. Description of progression | 8. Exercise description | 9. Home-exercise description | 10. Description of nonexercise components | 11. Adverse events | 12. Setting described | 13. Description of exercise interventions | 14a. Generic or tailored exercises | 14b. Description of tailored exercises | 15. Level of exercises (beginner, intermediate, advanced) | 16a. Measure of exercise adherence | 16b. Reporting of adherence | **Sum** |
| --- | --- | --- | --- | --- | --- | --- | --- | --- | --- | --- | --- | --- | --- | --- | --- | --- | --- | --- | --- | --- |
|  |  |  |  |  |  |  |  |  |  |  |  |  |  |  |  |  |  |  |  |  |
| Carpenter et al., 1995 | 1 | 0 | 1 | 1 | 1 | 0 | 0 | 0 | 1 | 1 | NA | 1 | 1 | 1 | 1 | 1 | 1^a^ | 1 | 1 | **14** |
| Friggi Sebe Petrelluzzi et al., 2012 | 0 | 1 | 1 | 1 | 1 | 1 | 0 | 0 | 0 | NA | 1 | 0 | 1 | 0 | 0 | 0 | 0 | 0 | 0 | **7** |
| Goncalves et al., 2017 | NA | 1 | 1 | 1 | 0 | 0 | 0 | 0 | 1 | NA | 1 | 1 | 1 | 1 | 0 | 0 | 1^a^ | 0 | 1 | **10** |
| Awad et al., 2017 | 1 | 0 | 1 | 1 | 1 | 0 | 0 | 0 | 1 | 1 | NA | 0 | 1 | 1 | 0 | 0 | 0 | 0 | 0 | **8** |
| Sum | **2** | **2** | **4** | **4** | **3** | **1** | **0** | **0** | **3** | **2** | **2** | **2** | **4** | **3** | **1** | **1** | **2** | **1** | **2** |  |

^a^ Inclusion criterion was nonregular exercisers
NA, not applicable
